# miR-497-5p Expression and Biological Activity in Gastric Cancer

**DOI:** 10.7150/jca.90087

**Published:** 2024-05-30

**Authors:** Xin Chen, Linlin Zhou, Yaqin Han, Suping Lin, Li Zhou, Wei Wang, Wei Zhang, Shihai Xuan, Jianxiu Yu, Wenjie Zheng

**Affiliations:** 1 Department of Medical Laboratory, Dongtai People's Hospital, Nantong University School of Medicine,Dongtai 224200, Jiangsu, P. R. China.; 2 Department of Oncology, Dongtai People's Hospital, Nantong University School of Medicine, Dongtai 224200, Jiangsu, P. R. China.; 3 Department of Medical Laboratory, Dongtai People's Hospital, Dongtai 224200, Jiangsu, P. R. China.; 4 Clinical Trial Center, Affiliated Hospital of Nantong University, Nantong 226001, P. R. China.

**Keywords:** miR-497-5p, gastric cancer, ERBB2, molecular, target

## Abstract

**Background:** This research aims to investigate the expression and biological roles of miR-497-5p in gastric cancer (GC), and its possible mechanisms.

**Methods:** Real Time Quantitative PCR (RT-qPCR) was performed to detect miR-497-5p in GC and normal tissues, as well as GC cell lines versus normal gastric mucosal cells (GES-1). The effects of miR-497-5p overexpression on proliferation were measured by the cell counting kit-8 (CCK8) assay and ethidium bromide (EdU) assay. Flow cytometry was used to assess the cell cycle. The migration and invasion were evaluated by scratch assay and Transwell assay, respectively. Gene targets of miR-497-5p were predicted using “multiMiR” R package combined with mirTarPathway database. And then luciferase reporter experiment was used to evaluate the activity of ERBB2 by miR-497-5p mimics in GC cell line. Besides, functional experiments were performed to verify the impact of miR-497-5p /ERBB2 on phenotypes of GC cells.

**Results:** Compared with the normal tissues and mucosal cells, miR-497-5p was reduced in GC tissues and GC cell lines. miR-497-5p significantly decreased proliferation, migration, and invasion capacity, with an elevated apoptosis ratio of gastric cancer cells. Bioinformatics indicated that ERBB2 might be the potential target of miR-497-5p Dual-luciferase reporter experiments showed it adversely regulated ERBB2 3'UTR luciferase activity. The expression of ERBB2 in GC tissues and cells is significantly higher compared to normal tissues and cells. Over-expression of ERBB2 in gastric cancer cells significantly reduced miR-497-5p's inhibitory effect on the malignant behavior of GC cells.

**Conclusion:** miR-497-5p was significantly down-regulated in GC tissues and cells, which inhibited the malignant features of GC cells by targeting ERBB2.

## Introduction

Gastric cancer is one of the common malignant tumors in the digestive system. Based on the data from the International Agency for Research on Cancer (IARC), there were approximately 1.089 million new cases of gastric cancer worldwide in 2020, placing it fifth in the incidence of malignancies. Additionally, gastric cancer accounted for around 769,000 deaths, ranking fourth among all malignant tumors in mortality 1. In China, gastric cancer exhibits the highest incidence rate among all malignant tumors affecting the digestive system 2. Most cases are already in the advanced stage when they are discovered. Most cases are already advanced at diagnosis, and the 5-year survival rate after surgical intervention is still less than 30% 3.

microRNA (miRNA) is an endogenous non-coding short RNA that has a significant role in inflammation, immunology, and tumor growth. Evidence suggests that miR-497-5p inhibits tumor cells in a variety of carcinogenesis settings, including melanoma, osteosarcoma, angiosarcoma, lung, pancreatic, and cervical malignancies 4-9. For pancreatic ductal adenocarcinoma (PDAC), Wong reported that miR-497-5p inhibited cancer cell progression by suppressing the oncogenic HOTTIP-HOXA13 pathway 6. Additionally, miR-497-5p has been confirmed to be involved in chemo-resistance for cisplatin, such as ovarian cancer, cervical cancer, and non-small-cell lung cancer 10-12. Besides this, in colorectal cancer, it has been reported that miR-497-5p can increase chemo-sensitivity to 5-fluorouracil treatment by targeting KSR1 13. However, the correlation between the miR-497-5p and gastric cancer has rarely been explored, which warrants further consideration.

In the present study, we focused on investigating the effects of miR-497-5p and its target genes on GC by bioinformatic methods and in vitro experiments. We explored the impact of miR-497-5p expression on cell proliferation, migration, and invasion for GC cells. In addition, we also found that ERBB2 could also be an important target of miR-497-5p in GC cells. It is supposed that miR-497-5p / ERBB2 axis contributes to the progression of GC.

## Materials and methods

### Main experimental materials

#### Gastric cancer tissue samples

In 2019 to 2022, 60 individuals with stomach cancer treated in Dongtai People's Hospital's gastrointestinal and oncology departments were chosen for the study. The inclusion criteria of the patients were as follows: 1) males or females aged ≥18 years; 2) clinically confirmed with TNM II or TNM Ⅳ through histology and/or cytology; 3) an ECOG performance status score of 0-1; 4) expected survival of ≥6 months. The exclusion criteria were as follows: 1) presence of immunodeficiency; 2) other malignant tumors progressing or requiring active treatment within the past 5 years (except for basal cell carcinoma or squamous cell carcinoma of the skin, or in situ cancer previously treated with potentially curative therapy), known allergies (grade ≥3) to any of the study drugs or excipients; 3) active autoimmune disease requiring systemic treatment (except for replacement therapy) within the past 2 years; 4) pregnancy or breastfeeding, or planning to become pregnant during the anticipated study duration; 5) known psychiatric or substance abuse disorders that would interfere with compliance to the study requirements 14. A detailed patient information table can be found in Table S1. This study was approved by the ethics committee of Dongtai People's Hospital and authorized the study. The informed permission was obtained from each patient. Gastric cancer cell lines and other experimental materials were provided as a separate attachment (Supplementary Materials).

### Experimental methods

#### Cell culture, transfection, and experimental grouping

The gastric cancer cell lines (AGS, NCI-N87, and HGC-27) and the normal gastric epithelial cell line (GES-1) were cultured in DMEM medium supplemented with 10% fetal bovine serum, maintaining a temperature of 37°C and an atmosphere of 5% CO_2_. Cells were cultured until they reached 80% fusion, then digested with 0.25% trypsin and passed on for further study. When cell growth reached 80% confluency, cell transfection was performed following the instructions of the Lipofectamine^TM^ 2000 reagent manual. The DMEM culture medium was replaced with serum-free and antibiotic-free DMEM culture medium 24 hours before transfection and the constructed the overexpressed miR-497-5p mimic lentiviral vector, pc-ERBB2 vector, and co-transfect the vector wtERBB2/miR-497-5p (wild-type ERBB2 gene) and mutERBB2/miR-497-5p (mutant ERBB2 gene), and miR-497-5p mimic + pc-ERBB2 were co- transfected., After 8 hours of transfection, replace the medium with a complete culture medium and continue incubation for 48 hours. Finally, collect cells for detecting transfection efficiency and conducting functional experiments. To inhibit DNA methyltransferase, the cells of HGC-27 were treated with the inhibitor 5-aza-2'-deoxycytidine (5-aza-dC) or dimethyl sulfoxide (DMSO) as a control.

#### RT-qPCR

TRIzol reagent is employed to extract complete RNA from tissues and cells. We performed reverse transcription to generate cDNA following the instructions of the reverse transcription kit. The reaction was conducted at 16°C for 30 minutes, followed by 42°C for another 30 minutes, and then heated to 85°C for 4 minutes. Reaction conditions: 10 minutes of 95°C pre-denaturation, 15 seconds of 95°C denaturation, 30 seconds of 60°C annealing, and 40 cycles of 72°C extension. Suzhou Jinweiji Biotechnology Co. CAGCAGCACACUGUGGUUUUGU-3 synthesized the miRNA-497-5p, ERBB2 (forward 5'-CGGACGCCTGATGGGTTAAT-3', and reverse 5'-AAAGGTTCTACCCCGCATGG-3') and U6 (forward 5'-CTCGCTTCGGCAGCACA-3', and reverse 5'-AACGCTTCACGAATTTGCGT-3') primers. The experiments were independently repeated 3 times and the average value was calculated.

#### Methylation-specific PCR (MSP)

The online database Methprimer (http://www.urogene.org/cgi-bin/methprimer/methprimer.cgi) was utilized to predict the CpG sites in the promoter region of miR-497-5p. DNA samples were subjected to bisulfite treatment using the EZ DNA Methylation-GoldTM Kit (D5006, Zyme Research). Methylation-specific PCR (MSP) analysis was performed using the GMS20024.2 MSP kit (GENMED). The primers for miR-497-5p MSP were custom designed and synthesized by Shanghai Shansan Biotechnology. The upstream primer (5'-3') for miR-497-5p MSP was AATTTTTTTGTTAGGATTTGATCGA, and the downstream primer (5'-3') was AATTTTAACCGTAACCCTATCTCGT. The upstream primer (5'-3') for miR-497-5p unmethylated MSP (UMSP) was AATTTTTTTGTTAGGATTTGATTGA, and the downstream primer (5'-3') was AATTTTAACCATAACCCTATCTCATC.

#### Chromatin Immunoprecipitation (ChIP)

The ChIP method was employed to validate the association between DNA methyltransferases (DNMT1, DNMT3a, and DNMT3b) and the promoter sequence of miR-497-5p. Firstly, DNA fragments were prepared from HGC-27 cells through sonication, followed by centrifugation at 13,000 × g for 10 minutes at 4°C. Immunoprecipitation was then performed using antibodies targeting DNMT1 (#5032, CST), DNMT3a (#49768, CST), and DNMT3b (#57868, CST). Subsequently, the DNA fragments bound by the above antibodies were purified using 80 µL of DNA purification suspension, and the specific primers targeting the CpG-enriched upstream region of miR-497-5p were used for RT-qPCR detection.

#### Scratch assay to detect cell migration ability

HGC-27 cells in each group were placed in 6-well plates at the density of 2 x 10^5^ cells/mL. Horizontal lines were drawn in each well with a marker pen and a ruler, generating 5 lines per well, evenly spaced every 1 cm. Cells were cultured under 5% CO_2_ at 37°C until they reached approximately 80% of confluency, following which the supernatant was extracted. A gun head was used to draw a horizontal scratch in each well, perpendicular to vertical lines on the back of the plate, and washed 3 times with PBS solution to remove any loose cells. After that, a serum-free DMEM culture medium was supplied, and cellular status was visually observed and recorded under a microscope at 0 h and 48 h.

#### Migration and invasion assay for Transwell

50 mg/L Matrigel gel (1:8 dilution) was equally distributed on the bottom of the upper chamber for the Transwell invasion assay. Next, the lower Transwell was filled with 600 μL of DMEM (100 mL/L fetal bovine serum). Lastly, 200 μL of each cell suspension (2 x 10^4^ cells) was administrated into the upper chamber. After 24 hours, the chambers were discarded and rinsed three times in PBS solution before being fixed in 40 g/L paraformaldehyde for 20 minutes and stained with 1 g/L crystal violet for 10 minutes. The migration experiment did not contain any stromal gel.

#### Dual luciferase reporter assay

HGC-27 cells were inoculated in 96-well plates (2 x 10^5^ cells) and cultured when the cell density was approximately 80%. The Lipofectamine ^TM^ 2000 transfection kit was used for transfection, and the co-transfected recombinant plasmids wtERBB2/miR-497-5p and mutERBB2/miR-497-5p were transfected into HGC-27 cells, respectively. As the control group, wtERBB2/NC miR-497-5p was employed. After 48 hours of incubation, gene expression was measured with a Luciferase Reporter Gene Assay Kit.

#### CCK8 assay to detect cell proliferation

Following transfection, HGC-27 cells were resuspended in DMEM media, and 2 x 10^4^ cells were seeded into 96-well plates. The plates were split into 3 groups, each with 5 replicate wells. After cell walling, cell activity was measured at 0h, 24h, 48h, and 72h according to the instructions of the CCK8 kit. 10μL of CCK8 working solution was added to each well for 2 h. The absorbance of each well was measured by a microplate reader at the wavelength of 450 nm.

#### EdU assay to detect cell proliferation

A 96-well plate was seeded with transfected HGC-27 cells. After incubation for 2 hours, 100μL of diluted EdU solution was added to each well. Samples were fixed in 4% paraformaldehyde for 30 minutes. The fixative was discarded, 2 mg/mL glycine was added, incubated for 5 minutes, and washed with PBS. The cells were incubated in a 0.5% TritonX-100 solution for 10 min, and sequentially added Apollo staining and Hoechst33342 reaction solution for 30 min, and washed with PBS. The cells were observed under a fluorescent microscope.

#### Western blot

Cells were harvested and subjected to SDS-PAGE. The proteins were separated according to molecular weight. Subsequently, the protein was transferred to PVDF membrane, followed by blockade with 5% milk for 1 h. ERBB2(ab134182, Abcam, UK) was probed with primary antibody. GAPDH (ab8245, Abcam, UK) was used as loading control.

#### Flow cytometry to detect apoptosis in each group

Following the transfection of HGC-27 cells for 48 hours, 0.25% trypsin was added to disperse the cells into a single-cell suspension. The resulting cells were centrifuged at 1000 rpm, resuspended in PBS, and adjusted to a cell concentration of 4×10^5^ cells/mL. The Annexin V-APC/7-AAD assay kit was used to detect the apoptosis rates of each group of cells by FACS Calibur flow cytometer according to the manufacturer's instructions. Each group was tested in triplicate.

#### Data processing and statistical analysis

The data are presented as mean ± standard deviation and analyzed using the SPSS 17.0 statistical software. Unpaired Student's t-test was utilized to compare two groups, whereas one-way ANOVA was employed to compare multiple groups. Statistical significance is indicated as follows: *P<0.05, #P<0.05, and $P<0.05 denote a significant difference, while **P<0.05, ##P<0.05, and $$P<0.05 denote an extremely significant difference.

## Results

### Expression of miR-497-5p in GC tissues and cells

**Figure 1:**
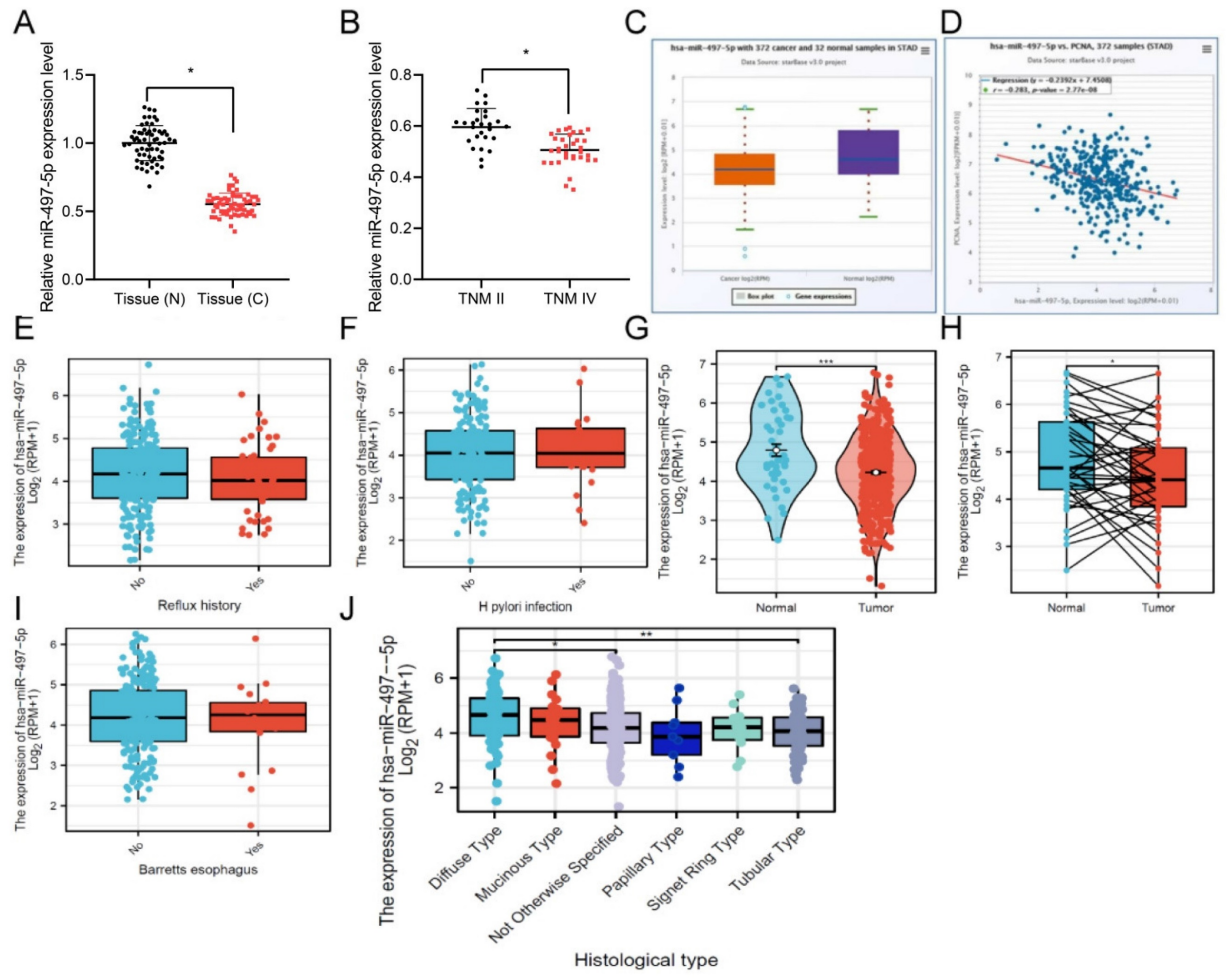
miR-497-5p expression in gastric cancer tissues and cells. A. qRT -PCR analysis of miR-497-5p in GC (n = 60) and normal tissues (n = 60). * * * P < 0.001. B. Expression of miR-497-5p in TNM stage II (n = 27) and TNM stage IV (n = 33) GC tissues * P < 0.05. C. Expression of miR-497-5p in gastric adenocarcinoma (n = 372) and normal tissues (n = 32) analyzed from the TCGA database. p < 0.001. D. Expression of miR-497-5p in gastric adenocarcinoma tissues analyzed from the TCGA database to analyze the correlation between miR-497-5p and PCNA mRNA expression in gastric adenocarcinoma tissues (n = 372). p < 0.001. E. The expression level of miR-497-5p in TCGA database with or without esophageal reflux history. F. The expression level of miR-497-5p in TCGA database with or without HP infection. G. The expression level of miR-497-5p in GC and non-paired normal tissues in the TCGA database, GC group vs. normal tissue ***P <0.001.H. The expression level of miR-497-5p in GC and paired normal tissues in the TCGA database, GC group vs. normal tissue **P <0.01. I. The expression level of miR-497-5p in Barretts esophagus in the TCGA database. J. The expression level of miR-497-5p in different histological types in the TCGA database. Diffuse type vs. not otherwise specified, *P<0.05; diffuse type vs. tubular type **P<0.01.

The expression features of miR-497-5p were evaluated in GC tissues and normal tissues. miR-497-5p was considerably lower in GC tissues compared to controls, as evidenced by reverse transcription quantitative polymerase chain reaction data (Fig. 1A). Similar results were observed in cell experiments, where the expression of miR-497 was found to be lower in gastric cancer cell lines (AGS-1, NCI-N87, and HGC-27) compared to normal gastric epithelial cell lines (GES-1) (Fig. S1A).

The GC population was divided into TNM II and TNM IV stages. Compared to TNM II stage tumor samples, miR-497-5p levels were noticeably lower in TNMIV tumor samples (Fig. 1B). This finding is consistent with the clinical data of the patients, indicating a negative correlation between the expression level of miR-497-5p and the clinical pathological grade of the patients (Table S1). Moreover, we used the TCGA database to validate miR-497-5p expression and showed low expression in cancer tissues (Fig. 1C). Using data from the TCGA, researchers found that miR-497-5p and the expression of the cancer marker PCNA were inversely correlated in patients with stomach cancer (Fig. 1D).

Moreover, our analysis of the TCGA database revealed that the expression level of miR-497-5p was not significantly associated with the presence of esophageal reflux, Helicobacter pylori (HP) infection or Barrett's esophagus. Nonetheless, we observed differences in the expression of miR-497-5p between paired and non-paired tumor and normal tissue samples (Fig. 1E, F, I). Notably, the expression of miR-497-5p was significantly downregulated in tumor tissues compared to normal tissues (Fig. 1G, H, P<0.05). Notably, significant differences were found in the expression of miR-497-5p among different histological types of gastric cancer, especially between diffuse-type and tubular-type gastric cancer (Fig. 1J, P<0.01).

Epigenetic dysregulation is a significant cause of abnormal miRNA expression in cancer. A previous study has suggested that high methylation of the upstream CpG island of miR-497-5p leads to its decreased expression in tumors 15. We predicted potential CpG islands within the promoter region of miR-497-5p using the online database MethPrimer (Fig. S1B) and designed specific methylation-specific PCR (MSP) primers to validate our predictions. MSP experimental results revealed the presence of methylation bands in gastric cancer tissues (Fig. S1C). Similar results were obtained when MSP experiments were conducted using normal epithelial cell lines and gastric cancer cell lines. Methylation was detected in the miR-497-5p promoter region in HGC-27 cells (Fig. S1D). Furthermore, treatment with the DNA methyltransferase inhibitor (5-Aza-dc) resulted in upregulation of miR-497-5p in HGC-27 cells (Fig. S1E), further confirming the regulatory role of DNA methylation in the expression of miR-497-5p. Additionally, exposure to 5-Aza-dc significantly reduced the binding of DNA methyltransferases (DNMT1, DNMT3a, and DNMT3b) to the promoter region of miR-497-5p (Fig. S1F). These results indicate that the downregulation of miR-497-5p in gastric cancer is attributed to the high methylation of its promoter CpG island.

### miR-497-5p inhibited the proliferation and induced apoptosis

**Figure 2:**
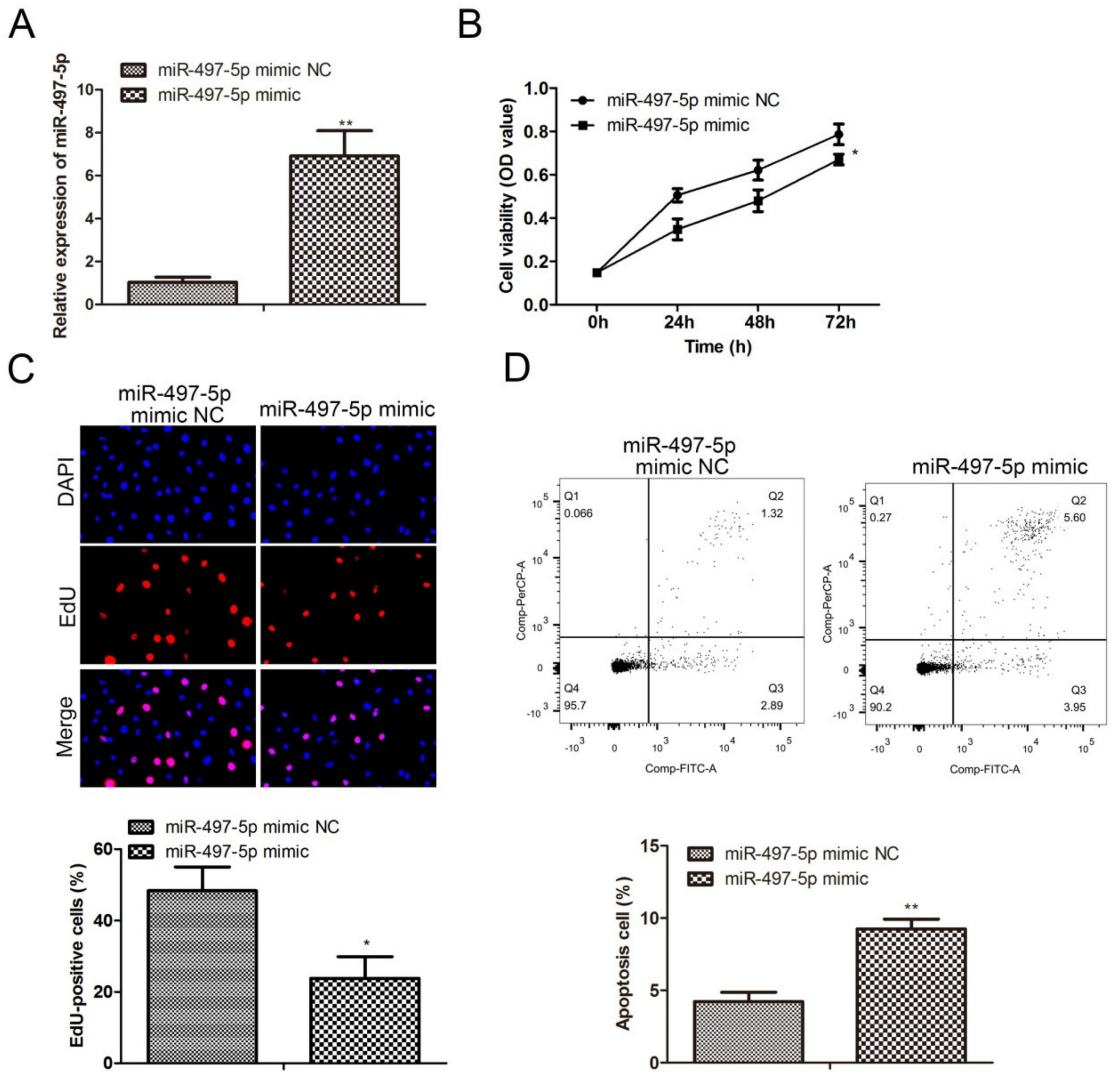
Effect of overexpression of miR-497-5p on proliferation and apoptosis of gastric cancer cell lines. A. RT-qPCR to detect the expression level of miR-497-5p in cells after transfection; B. CCK8 assay to detect cell proliferation; C. EdU assay to detect cell proliferation; D. Flow cytometry to detect cell apoptosis. miR-497-5p mimic group vs. miR-497-5p mimic NC group, *P < 0.001, **P < 0.01.

We cultured miR-497-5p-overexpressing cell line and its control cell line with HGC-27 (Fig. 2A), and the results indicated that miR-497-5p was dramatically increased in the transfected group compared to the miR-497-5p mimic NC group (*P<0.01*). Cell proliferation was measured using CCK8 and EdU assays, and we discovered that cells in the miR-497-5p mimic group had significantly reduced proliferation than cells in the miR-497-5p mimic NC group (*P<*0.0001) (Fig. 2B, C). When miR-497-5p mimic transfected cells were overexpressed, the proliferation of gastric cancer cells was halted. The flow cytometry results showed that the apoptosis rate of miR-497-5p mimic cells was significantly higher than that of miR-497-5p mimic NC cells (*P<0.01*) (Fig. 2D).

### miR-497-5p overexpression influences gastric cancer cell migration and invasion

**Figure 3:**
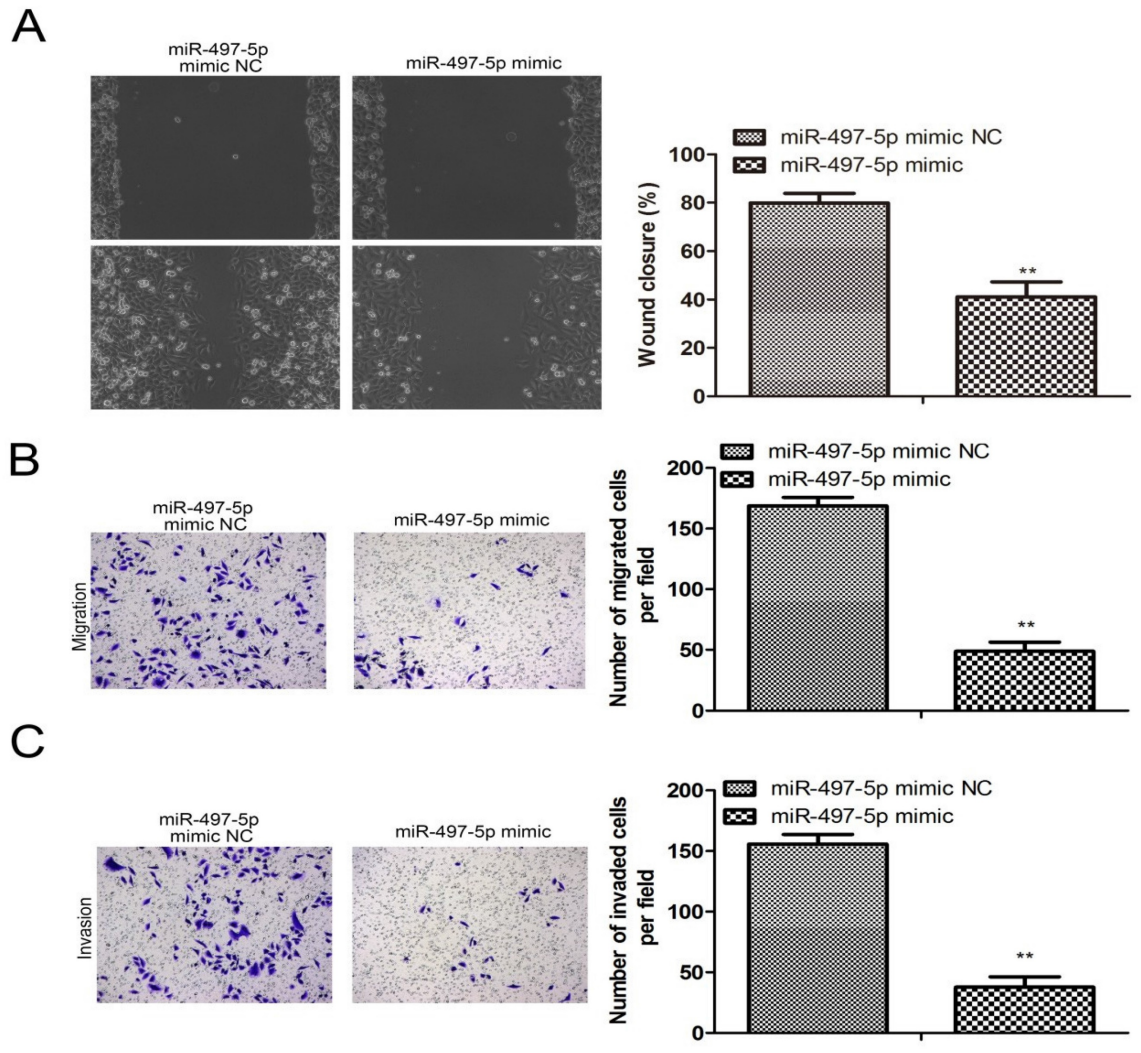
Effect of overexpression of miR-497-5p on migration and invasion of gastric cancer cell lines. a. Cell scratching assay; b. Transwell migration assay; c. Transwell invasion assay. miR-497-5p mimic group vs. miR-497-5p mimic NC group, **P < 0.01.

The results from the cell scratch assay are shown in Fig. 3A. Compared to the miR-497-5p mimic NC group, miR-497-5p-transfected HGC-27 cells showed significantly worse scratch healing (*P<0.01*). The number of HGC-27 cells that were able to penetrate the vesicle membrane was significantly lower in the miR-497-5p mimic NC group compared to the transwell assay results (*P<0*.01) (Fig. 3B, C). HGC-27 cell migration and invasion were suppressed by using a miR-497-5p mimic.

### Analysis of miR-497-5p target genes

**Figure 4:**
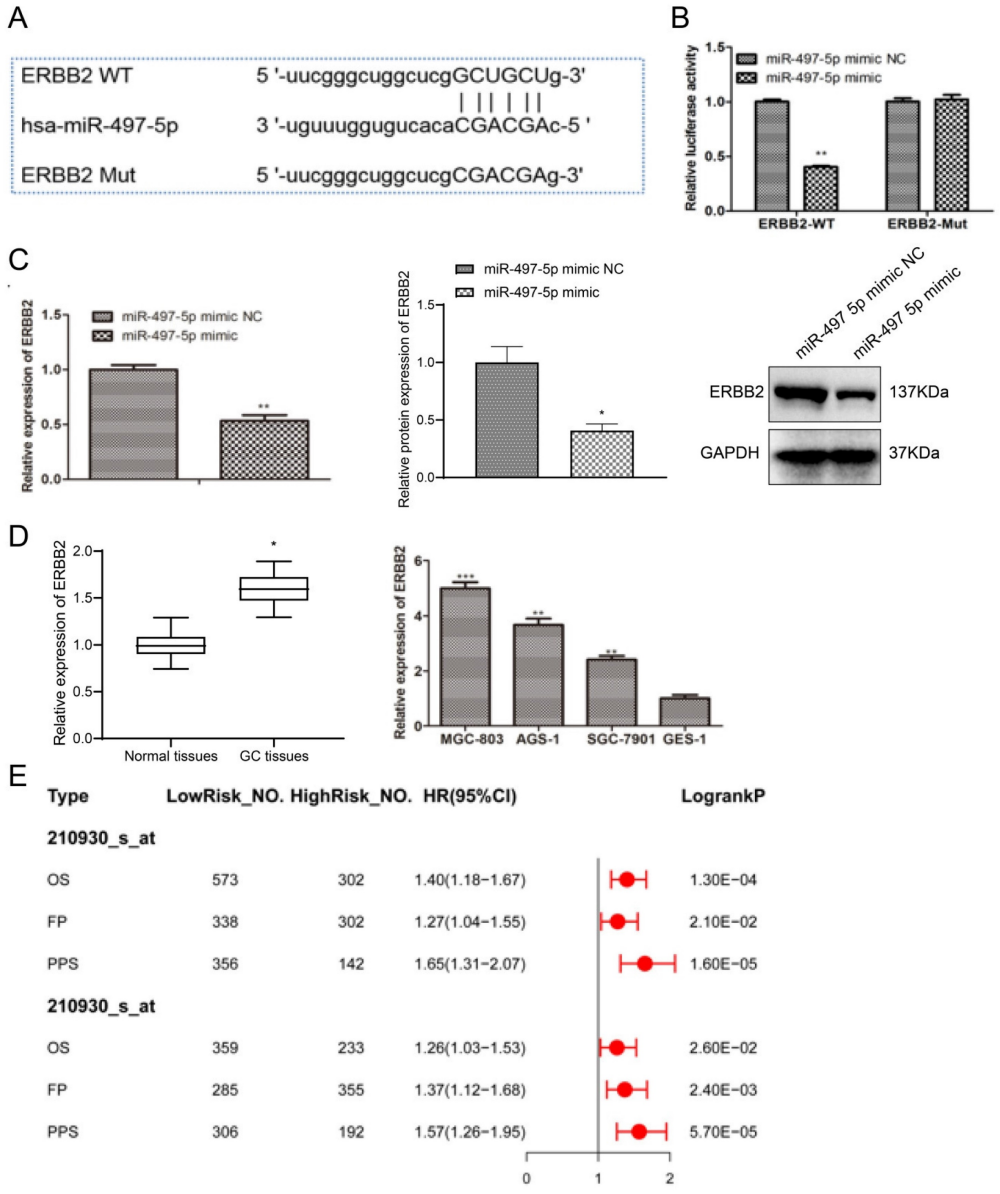
miR-497-5p targeting relationship with ERBB2 by bioinformatics. a. Bioinformatics analysis; b. Dual luciferase reporter assay; c. ERBB2 mRNA levels and protein levels in cells after transfection; d. RT-qPCR detection of ERBB2 expression levels in gastric cancer tissues versus normal tissues; e. RT-qPCR detection of ERBB2 expression levels in gastric cancer cells versus expression levels in normal cells. miR-497-5p mimic group vs. miR-497-5p mimic NC group, **P < 0.01; GC tissues group vs. Normal tissues group, *P < 0.05; AGS-1, NCI-N87 group vs. GES-1 group, **P < 0.01; HGC-27 group vs. GES-1 group***P < 0.001.

miRNAs may play oncogenic or tumor-suppressive roles which mainly depend on the biological roles of their target genes. Thus, on one hand, we used “multiMiR” R package to predict the target genes of miR-497-5p. On the other hand, considering single gene associations, gene-gene interactions, and gene-environment interactions, we also referred to the results from mirTarPathway database and the biological function of predicted target genes, and the results from mirTarPathway database showed that miR-497-5p was involved in pathways that regulate multiple cellular processes such as Wnt, Vegf, P53, and Erbb signaling pathway (Fig. S2 A). We obtained 3711 genes for miR-497-5p through “multiMiR” R package, and the details were listed in Table S2 (https://doi.org/10.6084/m9.figshare.24882231). Furthermore, KEGG pathway analysis showed that these target genes were involved in five significant biological process, such as choline cell resistance action, Breast Gastric mTor cells, AMPK signaling and cell adhesion signaling (Fig. S2 B). Given the results, we focused on the functional links between miR-497-5p and Erbb signaling pathway. ERBB2, the pivotal effector of the Erbb signaling pathway, was predicted in our results (Table S2 (https://doi.org/10.6084/m9.figshare.24882231)). Fig. 4A demonstrates that miR-497-5p could bind to the 3'-UTR of wild-type ERBB2. Given this evidence, we hypothesized that miR-497-5p targets ERBB2. Mimics of miR-497-5p strongly suppressed luciferase activity of wild-type ERBB2-3'-UTR (*P < 0.01*) but had no effect on ERBB2-3'-UTR mutants in dual-luciferase reporter studies (Fig. 4B), demonstrating direct action of miR-497-5p on ERBB2. When compared to the miR-497-5p mimic group, the RT-qPCR experiment demonstrated that the miR-497-5p mimic significantly reduced ERBB2 expression in gastric cancer cells (*P < 0.01*) (Fig. 4C). Analysis of ERBB2 expression characteristics in gastric cancer tissues revealed that ERBB2 expression levels were considerably higher than in normal tissues (*P < 0.05*) (Fig. 4D). The mRNA expression levels of ERBB2 were significantly higher in HGC-27, AGS-1, and NCI-N87 cells compared to GES-1 cells (*P < 0.01*) (Fig. 4E). Hence, ERBB2 may be a miR-497-5p target gene, significantly expressed in gastric cancer. For a prognostic point of view, data from the Kaplan-Meier plotter database showed that higher expression of ERBB2 was closely related to the poor prognosis of GC patients.

### miR-497-5p regulates gastric cancer cell activity by targeting ERBB2

**Figure 5:**
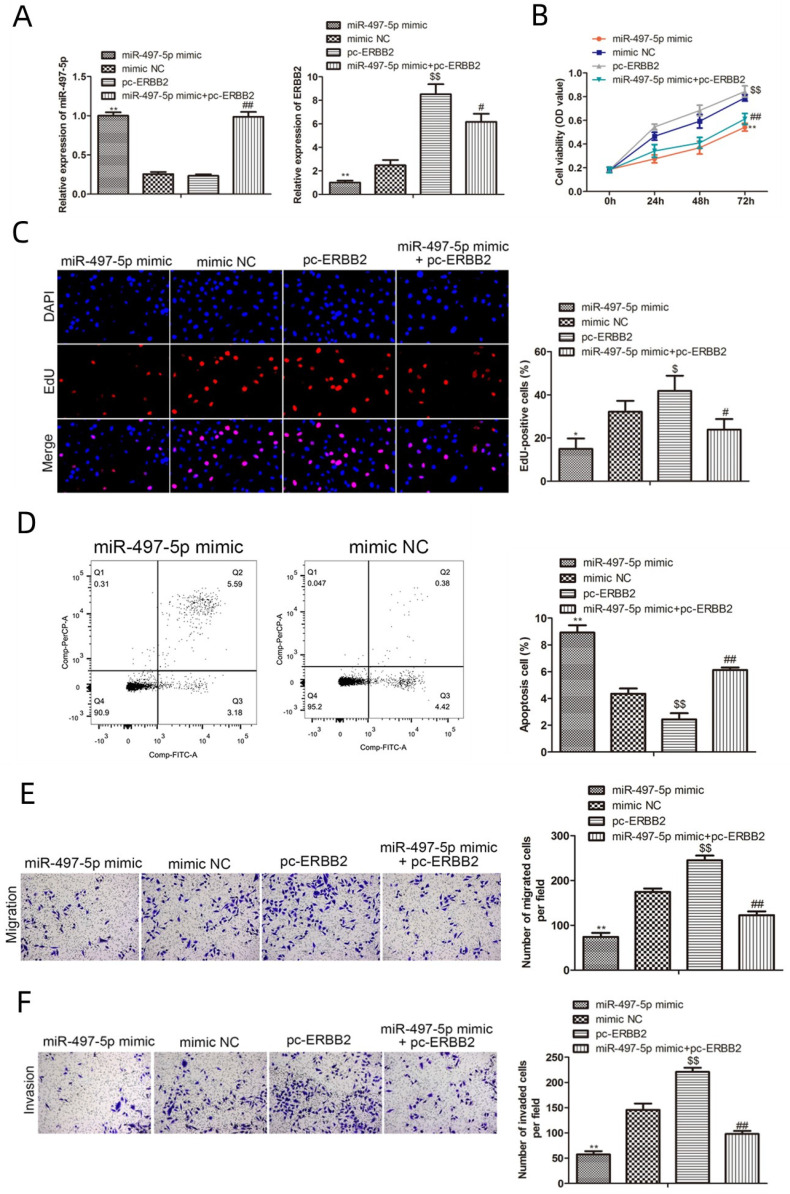
Functional rescue assays. A. RT-qPCR for miR-497-5p and ERBB2 expression; B. CCK8 assay for cell proliferation; C. EdU assay for cell proliferation; D. Flow cytometry for apoptosis; E. Transwell assay for cell migration; F. Transwell assay for cell invasion. miR-497-5p mimic group vs. mimic group, *P < 0.05, **P < 0.01; miR-497-5p mimic + pc-ERBB2 group vs. pc-ERBB2 group, #P < 0.05, #P < 0.01; pc-ERBB2 group vs. mimic group, $P < 0.05, $P < 0.01.

We performed rescue studies to investigate the role of miR-497-5p in the biological activity of gastric cancer cells, as ERBB2 is a potential target of this miRNA (Fig. 5A). The results from CCK8 and EdU experiments revealed that cell proliferation was significantly lower in miR-497-5p mimics group and miR-497-5p mimics with pc-ERBB2 group. Conversely, the cell proliferation was significantly higher in pc-ERBB2 group (Fig. 5B, C). These results demonstrated that miR-497-5p was able to reverse the biological function of ERBB2. The results of Transwell assay showed that the number rate of migration and invasion of miR-497-5p mimic cells was much lower than that of the mimic group (*P < 0.01*), while the apoptosis rate was significantly greater (*P < 0.01*) in the mimic group (*P < 0.01*). The number of cells migrating and invading was significantly higher in the miR-497-5p mimic+pc-ERBB2 group (*P < 0.01*) (Fig. 5E, F).

## Discussion

Expression repression is caused by miRNAs at the post-transcriptional level when they bind to the 3'UTR of their target mRNAs. Conversely, dysfunctional miRNAs prevent target genes for either tumor suppressors or oncogenes from being expressed, which is important for cancer pathogenesis. Thus, many miRNAs are down- or up-regulated in human cancers and function as oncogenic or tumor suppressor factors. miRNAs regulate solid tumourigenesis and metastasis by two main mechanisms: miRNAs regulate the level of DNA methylation and histone modifications, among other things, to influence the expression of genes involved in cellular growth and apoptosis. Moreover, miRNAs regulate the degree of DNA methylation or histone modification, affecting gene expression in cell growth and death.

Many studies have shown that miRNA dysregulation is important in the pathogenesis of GC 16. miRNAs can operate as oncogenes or tumor suppressors by regulating gene expression and controlling GC cell motility, invasion, and proliferation 12, 17-19. As a member of the microRNAs, miR-497-5p plays a key function in the genesis of cancer 20. miR-497-5p inhibits lung squamous cell carcinoma development by targeting CDCA4 21. miR-497-5p inhibits the malignant features of colorectal cancer cells by upregulating PTPN3 22. By targeting SOX5 in non-small-cell lung cancer, miR-497-5p suppresses tumour cell proliferation and invasion 12. miR-497-5p promotes apoptosis in ovarian cancer by regulating MTDH 12. Several studies have demonstrated that miRNA-497 affects the expression of specific target genes, hence contributing to the development and progression of a number of malignancies. Unfortunately, less research has been conducted on the effects of miR-497-5p on GC cells. According to the TCGA database, low miR-497-5p expression in stomach cancer patients was related with a poor prognosis. This study demonstrated that the expression of miR-497-5p was much lower in GC tissues than in normal tissues, corroborating the findings of a database analysis. While miR-497-5p expression levels were unexpectedly low in gastric cancer cell lines, overexpression of this microRNA could dramatically reduce gastric cancer cell proliferation, migration, and invasion. It was discovered that miR-497-5p inhibits the activity of gastric cancer cells but is only faintly expressed in this condition.

Expression of target genes can be silenced by miRNA binding to their 3'-UTR 23. It was hypothesized from this study's bioinformatic analyses that miR-497-5p would bind to the 3'-UTR of the ERBB2 gene. The dual luciferase reporter system test and RT-qPCR results demonstrated that ERBB2 was the miR-497-5p target gene in gastric cancer cells. ERBB2 gene is a proto-oncogene that, when overexpressed, is closely associated with gastric carcinogenesis and progression. The higher its expression, the less sensitive it is to chemotherapy and the more likely it is to recur and metastasis 24, 25. Higher levels of expression correlate with resistance to treatment and increased recurrence and metastasis risk. Consistent with other research, our results demonstrated that ERBB2 expression was much higher in GC tissues and cells than in normal tissues and cells 26, 27. ERBB2 can be involved in the biological process of tumors in lung, breast, and ovarian cancers 28-30. miR-497-5p inhibited the growth and metastasis of gastric cancer cells. However, ERBB2 significantly reversed this impact. Targeting ERBB2 implies that miR-497-5p may affect the biological function of gastric cancer cells.

Additionally, several other studies have revealed the multifaceted role of miR-497-5p in the pathogenesis of gastric cancer. Feng *et al.* demonstrated that miR-497-5p inhibits the proliferation and growth of gastric cancer cells by targeting PDK3 31. This finding underscores the importance of miR-497-5p in regulating cellular metabolic pathways. Similarly, Song *et al.* found that miR-497-5p suppresses ATG14, thereby modulating the chemoresistance of gastric cancer cells 32, further highlighting its potential application in gastric cancer treatment. Moreover, Tang *et al.* discovered that miR-497-5p improves the migration, invasion, and EMT of hypoxia-induced gastric cancer cells by downregulating the EGFR signaling pathway 33, revealing its role in regulating the tumor microenvironment. Wei *et al.* found that miR-497-5p regulates the cell cycle of gastric cancer cells by directly targeting CDC25A 34, demonstrating its importance in cell cycle control. Additionally, Xia *et al.* discovered that miR-497-5p inhibits the growth and promotes the apoptosis of gastric cancer cells by directly suppressing PIK3R1 35, further emphasizing its critical role in regulating tumor cell growth and death. Previous studies have demonstrated the significant role of miR-497-5p in the occurrence and development of gastric cancer through various signaling pathways 31-33, 35. This study innovatively integrated multiple database analyses, including TCGA, and conducted in vitro experiments to reveal and validate, for the first time, that the downregulation of miR-497-5p in GC inhibits the malignant transformation of GC cells by targeting ERBB2.

However, our study is the first to demonstrate that miR-497-5p specifically promotes the mRNA degradation of ERBB2, thereby inhibiting the expression of ERBB2 protein and suppressing the malignant behavior of gastric cancer cells. This finding not only provides new insights into the role of miR-497-5p in gastric cancer but also enriches the molecular pathogenesis of gastric cancer. These relevant findings offer potential new targets for the clinical diagnosis and treatment of gastric cancer, opening up new avenues for future research and therapeutic strategies.

Although this study has made significant progress in elucidating the role of miR-497-5p in the pathogenesis of gastric cancer, we must acknowledge an important limitation. The sample size in this study is relatively small, consisting of only 60 gastric cancer patients from the Gastroenterology and Oncology Departments of Dongtai People's Hospital. The limited sample size may restrict the generalizability and statistical power of our research findings, suggesting that our discoveries may not be widely applicable to larger and more diverse patient populations. Additionally, studies with small sample sizes are prone to be affected by chance variation, which can result in biases in research outcomes. However, in our study, there was a limited focus on clinical sample research, and therefore, the inclusion of 30 cases for small-scale experiments is sufficient to support our findings. Therefore, while the findings of this study have theoretical significance, larger-scale research is necessary to validate and expand upon these preliminary results before applying them to a broader population of gastric cancer patients.

In conclusion, our study provides new insights into the role of miR-497-5p in gastric cancer. However, further research, especially involving larger sample sizes, is crucial to establish the clinical significance of these findings. This will be instrumental in better assessing the effectiveness and applicability of miR-497-5p as a potential therapeutic target for gastric cancer. The results demonstrate that miR-497-5p is down-regulated in gastric cancer, and this down-regulation correlates with the prognosis. In gastric cancer, the fact that miR-497-5p inhibits GC cell motility, invasion, and proliferation by targeting ERBB2 suggests a potential theoretical basis and therapeutic target. These findings underscore the need for more extensive studies to validate and expand our understanding of miR-497-5p's role, which could pave the way for innovative treatments for gastric cancer.

## Supplementary Material

Supplementary figures and table.

## References

[1] Zurleni T, Gjoni E, Altomare M, Rausei S (2018). Conversion surgery for gastric cancer patients: A review. World J Gastrointest Oncol.

[2] Einama T, Abe H, Shichi S, Matsui H, Kanazawa R, Shibuya K (2017). Long-term survival and prognosis associated with conversion surgery in patients with metastatic gastric cancer. Mol Clin Oncol.

[3] Jemal A, Bray F, Center MM, Ferlay J, Ward E, Forman D (2011). Global cancer statistics. CA Cancer J Clin.

[4] Chai L, Kang XJ, Sun ZZ, Zeng MF, Yu SR, Ding Y (2018). MiR-497-5p, miR-195-5p and miR-455-3p function as tumor suppressors by targeting hTERT in melanoma A375 cells. Cancer Manag Res.

[5] Sun Z, Li A, Yu Z, Li X, Guo X, Chen R (2017). MicroRNA-497-5p Suppresses Tumor Cell Growth of Osteosarcoma by Targeting ADP Ribosylation Factor-Like Protein 2. Cancer Biother Radiopharm.

[6] Chen Y, Kuang D, Zhao X, Chen D, Wang X, Yang Q (2016). miR-497-5p inhibits cell proliferation and invasion by targeting KCa3.1 in angiosarcoma. Oncotarget.

[7] Hu W, Fan H, Chen T, Wang Y, Liu S (2021). Bioinformatics analysis of miR- 497 and its expression in lung adenocarcinoma. Journal of Modern Oncology.

[8] Dawei J, Liu XU (2020). miR-497-5p inhibits proliferation of pancreatic cancer cells by targeting CCNE1 gene. Cancer Research on Prevention and Treatment.

[9] Zhang J, Gao L, Zhan P, Mao X (2017). Inhibitory effect of miR-497-5p on proliferation of human cervical carcinoma cell. Shandong Medical Journal.

[10] Xu S, Fu GB, Tao Z, OuYang J, Kong F, Jiang BH (2015). MiR-497 decreases cisplatin resistance in ovarian cancer cells by targeting mTOR/P70S6K1. Oncotarget.

[11] Yang H, Wu X-L, Wu K-H, Zhang R, Ju L-L, Ji Y (2016). MicroRNA-497 regulates cisplatin chemosensitivity of cervical cancer by targeting transketolase. American journal of cancer research.

[12] Li G, Wang K, Wang J, Qin S, Sun X, Ren H (2019). miR-497-5p inhibits tumor cell growth and invasion by targeting SOX5 in non-small-cell lung cancer. J Cell Biochem.

[13] Wang L, Jiang CF, Li DM, Ge X, Shi ZM, Li CY (2016). MicroRNA-497 inhibits tumor growth and increases chemosensitivity to 5-fluorouracil treatment by targeting KSR1. Oncotarget.

[14] Bang YJ, Van Cutsem E, Fuchs CS, Ohtsu A, Tabernero J, Ilson DH (2019). KEYNOTE-585: Phase III study of perioperative chemotherapy with or without pembrolizumab for gastric cancer. Future Oncol.

[15] Peng Z, Zhang Y, Shi D, Jia Y, Shi H, Liu H (2021). miR-497-5p/SALL4 axis promotes stemness phenotype of choriocarcinoma and forms a feedback loop with DNMT-mediated epigenetic regulation. Cell Death Dis.

[16] Shin VY, Chu KM (2014). MiRNA as potential biomarkers and therapeutic targets for gastric cancer. World J Gastroenterol.

[17] Lin S, Gregory RI (2015). MicroRNA biogenesis pathways in cancer. Nat Rev Cancer.

[18] Svoronos AA, Engelman DM, Slack FJ (2016). OncomiR or Tumor Suppressor?. The Duplicity of MicroRNAs in Cancer. Cancer Res.

[19] van Kouwenhove M, Kedde M, Agami R (2011). MicroRNA regulation by RNA-binding proteins and its implications for cancer. Nat Rev Cancer.

[20] Xu GS, Li ZW, Huang ZP, Brunicardi FC, Jia F, Song C (2019). MiR-497-5p inhibits cell proliferation and metastasis in hepatocellular carcinoma by targeting insulin-like growth factor 1. Mol Genet Genomic Med.

[21] Hu J, Xiang X, Guan W, Lou W, He J, Chen J (2021). MiR-497-5p down-regulates CDCA4 to restrains lung squamous cell carcinoma progression. J Cardiothorac Surg.

[22] Hong S, Yan Z, Wang H, Ding L, Bi M (2019). Up-regulation of microRNA-497-5p inhibits colorectal cancer cell proliferation and invasion via targeting PTPN3. Biosci Rep.

[23] Yang N, Zhu S, Lv X, Qiao Y, Liu YJ, Chen J (2018). MicroRNAs: Pleiotropic Regulators in the Tumor Microenvironment. Front Immunol.

[24] Yano T, Ohtsu A, Boku N, Hashizume K, Nakanishi M, Ochiai A (2006). Comparison of HER2 gene amplification assessed by fluorescence in situ hybridization and HER2 protein expression assessed by immunohistochemistry in gastric cancer. Oncology reports.

[25] Ferreira D, Soares M, Correia J, Adega F, Ferreira F, Chaves R (2019). Assessment of ERBB2 and TOP2alpha gene status and expression profile in feline mammary tumors: findings and guidelines. Aging (Albany NY).

[26] Yuan S, Hua Y, Wang F, Liu J, Yuan Z (2012). Association of Her-2/neu expression and prognosis of gastric cancer. Jiangsu Medical Journal.

[27] Pei B, Cun YL (2015). Effect of Her-2 overexpression on the prognosis of gastric cancer and its clinical application. Journal of Modern Oncology.

[28] Li BT, Ross DS, Aisner DL, Chaft JE, Hsu M, Kako SL (2016). HER2 Amplification and HER2 Mutation Are Distinct Molecular Targets in Lung Cancers. J Thorac Oncol.

[29] Press MF, Sauter G, Buyse M, Fourmanoir H, Quinaux E, Tsao-Wei DD (2016). HER2 Gene Amplification Testing by Fluorescent In Situ Hybridization (FISH): Comparison of the ASCO-College of American Pathologists Guidelines With FISH Scores Used for Enrollment in Breast Cancer International Research Group Clinical Trials. J Clin Oncol.

[30] Chiu HH, Chao WR, Chen CK, Lee YJ, Lee MY, Han CP (2020). The Her2 gene aberrations in mucinous ovarian carcinoma: Analysis of twenty-one cases. Taiwan J Obstet Gynecol.

[31] Feng L, Cheng K, Zang R, Wang Q, Wang J (2019). miR-497-5p inhibits gastric cancer cell proliferation and growth through targeting PDK3. Biosci Rep.

[32] Song Z, Jia N, Li W, Zhang XY (2020). LINC01572 Regulates Cisplatin Resistance in Gastric Cancer Cells by Mediating miR-497-5p. Onco Targets Ther.

[33] Tang J, Zhu H, Lin J, Wang H (2021). Knockdown of Circ_0081143 Mitigates Hypoxia-Induced Migration, Invasion, and EMT in Gastric Cancer Cells Through the miR-497-5p/EGFR Axis. Cancer Biother Radiopharm.

[34] Wei W, Mo X, Yan L, Huang M, Yang Y, Jin Q (2020). Circular RNA Profiling Reveals That circRNA_104433 Regulates Cell Growth by Targeting miR-497-5p in Gastric Cancer. Cancer Manag Res.

[35] Xia TF, Chen J, Wu K, Zhang J, Yan Q (2019). Long noncoding RNA NEAT1 promotes the growth of gastric cancer cells by regulating miR-497-5p/PIK3R1 axis. Eur Rev Med Pharmacol Sci.

