# Supplementary material for: miR-497-5p Expression and Biological Activity in Gastric Cancer
Source: J Cancer. 2024 May 30;15(12):3995–4006. doi: 10.7150/jca.90087 (PMC11190777; doi:10.7150/jca.90087)
Supplement: Supplementary file 1 — Supplementary figures and table. [file jcav15p3995s1.pdf]

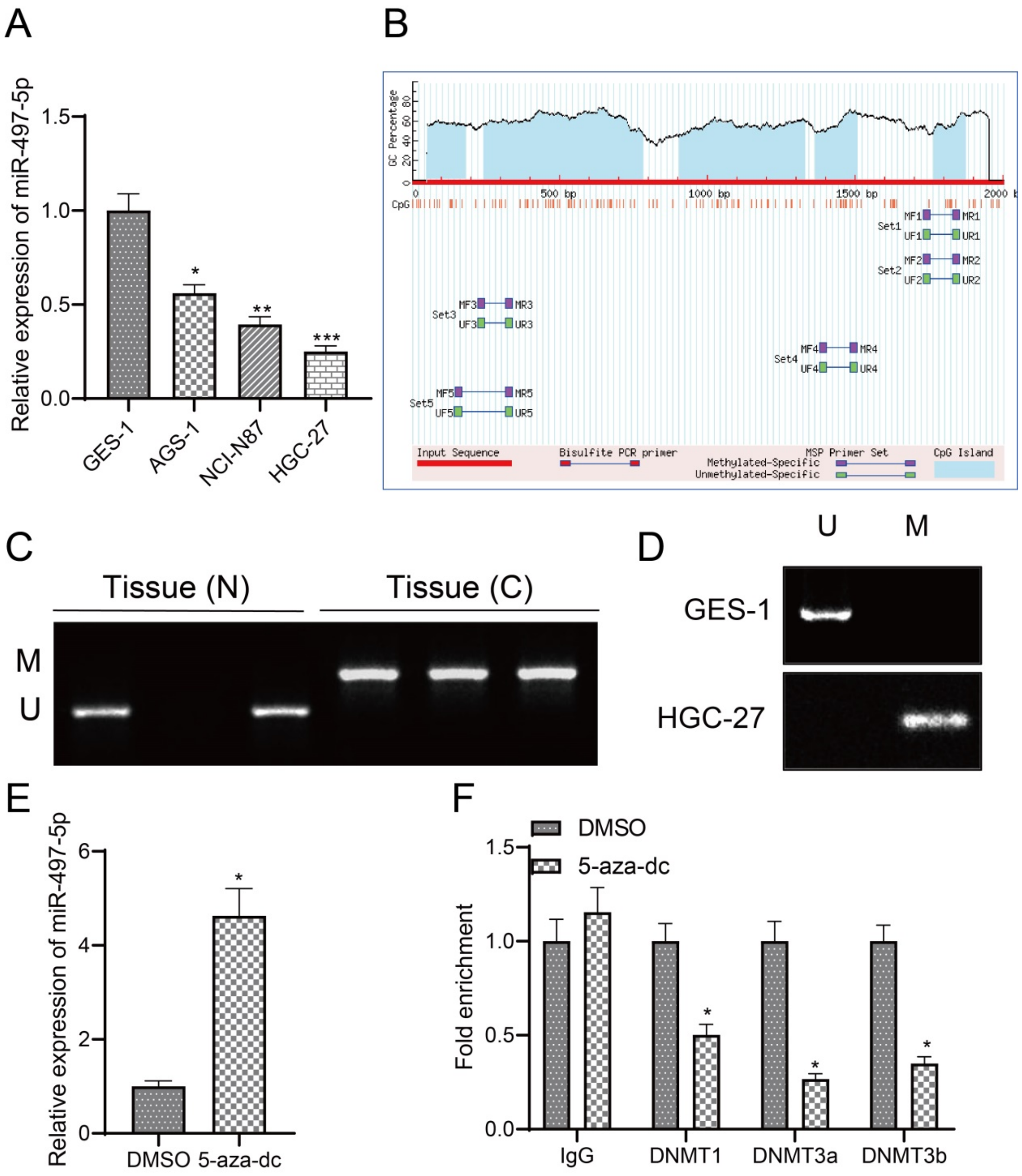

Fig. S1. High methylation induces downregulation of miR-497-5p in gastric cancer. A. Expression of miR-497-5p in normal gastric epithelial cells and gastric cancer cells determined by RT-qPCR. B. Prediction of the presence of a CpG island (highlighted in blue) upstream of the miR-497-5p promoter using the MethPrimer database. C. Methylation status of the promoter in gastric cancer tissues and normal tissues assessed by MSP. D. Methylation status of the promoter in normal gastric epithelial cells and gastric cancer cells determined by MSP. E. Expression of miR-497-5p in HGC-27 cells treated with 5-Aza-dc measured by RT-qPCR. F. ChIP experiments examining the interaction between

DNMT1/DNMT3 (a/b) and the miR-497-5p promoter. AGS-1 and NCI-N87 groups vs. GES-1 group, $P < 0.01$; HGC-27 group vs. GES-1 group, $P < 0.05$, $P < 0.001$; DMSO group vs. 5-Aza-dc group, $^*P < 0.05$.

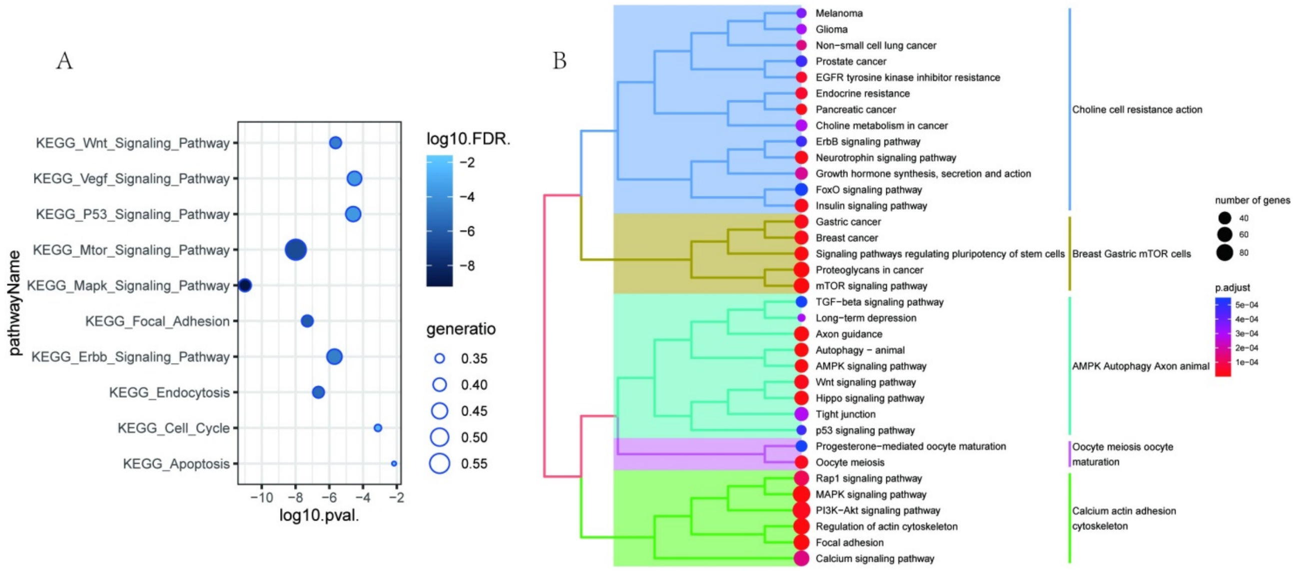

Fig. S2 A： mirTarPathway database and the biological function of predicted target genes；B： KEGG pathway analysis of target genes.

Table S1: Correlation between expression level of miR-49-5p and clinical pathology.

Clinical feature	n	miR-497-5p expression	p
Age			0.399
≤45	27	0.543 ± 0.073	
>45	33	0.561 ± 0.088	
Gender			0.895
Female	27	0.555 ± 0.067	
Male	33	0.552 ± 0.093	
Drinking			0.492
Yes	32	0.560 ± 0.088	
No	28	0.545 ± 0.074	
Smoking			0.116
Yes	35	0.567 ± 0.087	
No	25	0.534 ± 0.071	
Gastric ulcer			0.804
Yes	36	0.551 ± 0.063	
No	24	0.557 ± 0.104	
Duodenal ulcer			0.059
Yes	32	0.535 ± 0.075	
No	28	0.574 ± 0.085	
Stage			0.0001
II	27	0.596 ± 0.073	
IV	33	0.506 ± 0.062	
